# Sex modifies the association between insulin resistance and psychiatric comorbidities among individuals with type 2 diabetes

**DOI:** 10.1186/s12888-026-08502-2

**Published:** 2026-08-11

**Authors:** María Teresa Alcántara-Garcés, Alejandra Monserrat Estefanía Rodríguez-Ramírez, Victoria Landa-Anell, Marco Antonio Melgarejo-Hernández, Ana Cristina García-Ulloa, Sergio Hernández-Jiménez

**Affiliations:** 1 Centro de Atención Integral del Paciente con Diabetes (CAIPaDi), Instituto Nacional de Ciencias Médicas y Nutrición Salvador Zubirán, Vasco de Quiroga No. 15, Colonia Sección XVI, Tlalpan, Mexico City, Mexico; 2 Escuela de Medicina y Ciencias de la Salud, Tecnológico de Monterrey, Monterrey, 64710 México

**Keywords:** Type 2 diabetes, Insulin resistance, Sex differences, Psychiatric disorders, Anxiety, METS-IR

## Abstract

**Introduction:**

The co-occurrence of neuropsychiatric disorders and type 2 diabetes (T2D) is well-documented. Insulin resistance (IR) has emerged as a potential biological mechanism associated with psychiatric disorders. However, the influence of sex on this relationship remains underexplored. This study aimed to determine whether sex modifies the association between insulin resistance, assessed using the METS-IR Index, and psychiatric comorbidities in patients with type 2 diabetes.

**Methods:**

Comparative cross-sectional study of 4,699 individuals (2689 females, 2010 males) with T2D (duration < 10 years) who attended a multidisciplinary care center in Mexico City. IR was estimated using the METS-IR index (cutoff ≥ 50). Psychiatric comorbidities, including affective, anxiety, and eating disorders, were evaluated using standardized clinical assessments. Multiple logistic regression models were constructed to evaluate the association between the sex-insulin resistance interaction and four distinct psychopathological outcomes.

**Results:**

Females exhibited a significantly higher prevalence of all psychiatric comorbidities compared to males: affective (27.8% vs. 13.5%), anxiety (25.9% vs. 17.5%), and eating disorders (28.9% vs. 20.1%) (all *p* < 0.001). In females, high IR (METS-IR ≥ 50) was significantly associated with a higher frequency of affective disorders (30.5% vs. 26.0%, *p* = 0.011), anxiety disorders (28.0% vs. 24.5%, *p* = 0.040), and eating disorders (38.3% vs. 22.7%, *p* < 0.001). Conversely, in males, high METS-IR was only associated with eating disorders (27.0% vs. 14.4%, *p* < 0.001), showing no significant impact on affective or anxiety symptoms. Females with METS-IR ≥ 50 were two-fold more likely to have any psychiatric disorder (OR = 2.40; IC95%:1.85–3.11).

**Conclusion:**

Higher METS-IR was associated with psychiatric comorbidities in a sex-specific manner. These findings suggest that the relationship between insulin resistance and psychiatric disorders differs between females and males. While IR broadly increases the risk for multiple psychiatric domains in females, its impact in males is primarily restricted to eating behaviors. These findings underscore the critical need for sex-stratified metabolic monitoring to improve mental health screening and integrated care in T2D.

## Introduction

The co-occurrence of neuropsychiatric disorders and type 2 diabetes (T2D) has been frequently reported. Patients with T2D report a higher prevalence of depressive, anxiety, eating, sleep, psychotic, and neurocognitive disorders than the general population. T2D is also associated with increased risk of mood disorders and dementia [1]. The link between the mind and metabolic disorders is bidirectional and complex; these comorbidities share several pathways [2]. Peripheral insulin resistance is a hallmark of type 2 diabetes and has emerged as a potential biological mechanism linking metabolic dysfunction with psychiatric disorders. Although clinical studies typically rely on surrogate indices of peripheral insulin resistance, accumulating evidence suggests that peripheral metabolic dysfunction is associated with alterations in brain insulin signaling and neuropsychiatric outcomes. Researchers have reported failures in insulin signaling that affect brain metabolism, neurotrophism, neuroplasticity, and neuromodulation [3–5].

Insulin resistance is defined as an impaired biological response to insulin in classical peripheral target tissues, including the liver, skeletal muscle, and adipose tissue. Increasing evidence also indicates that peripheral insulin resistance is associated with altered insulin signaling within the central nervous system, providing a potential biological link between metabolic dysfunction and neuropsychiatric disorders [3, 6]. Because direct assessment of insulin resistance using the hyperinsulinemic-euglycemic clamp or even surrogate measures such as HOMA-IR is impractical in large clinical cohorts, validated surrogate indices have become valuable tools for epidemiological research. The Metabolic Score for Insulin Resistance (METS-IR) is a non–insulin–based index derived from fasting glucose, triglycerides, HDL cholesterol, and body mass index that has demonstrated good agreement with established measures of insulin sensitivity and has been validated for identifying insulin resistance in the Mexican population. Although METS-IR reflects peripheral rather than central insulin resistance, growing evidence suggests that peripheral metabolic dysfunction is closely linked to alterations in brain insulin signaling and neuropsychiatric outcomes. Therefore, METS-IR provides a clinically relevant and feasible surrogate marker for investigating the relationship between insulin resistance and psychiatric comorbidities in patients with type 2 diabetes. The insulin receptor is present throughout the brain and plays important roles in whole-body metabolism and CNS function [7]. Altered insulin signaling within the CNS has been associated with changes in dopaminergic signaling, neuroplasticity, brain development, blood-brain barrier function, and mitochondrial function, mediated by insulin receptors in neurons and glial cells [8–10]. These alterations affect central processes of emotional regulation, orexigenic/anorexigenic pathways, the reward system, inhibitory control, motivation, and memory preservation, among other neurocognitive functions [11, 12]. Various studies of large genetic and metabolic cohorts, neuronal biomarkers, neuroimaging, and intervention trials with insulin sensitizers have reported that insulin resistance in the CNS is closely linked to depressive symptoms, cognitive impairment, and a worse course of psychiatric conditions [13–16].

Affective disorders, anxiety, and eating disorders are among the most frequent psychiatric comorbidities of T2D, which have been proposed as part of the insulin resistance phenotype [5]. These disorders are more prevalent in females, both in T2D and in the general population. Various characteristics have been proposed to explain these disparities, including psychosocial factors, sex hormones, and genetics [17–19]. Nonetheless, the role of insulin resistance has not been explored in this manner. Previous research reported sex differences in insulin sensitivity and resistance, with greater effects in postmenopausal females [20]. Nonetheless, the evidence on the clinical correlates of insulin resistance by sex in patients with T2D and psychiatric disorders is still limited. This study investigated whether sex modifies the association between insulin resistance and psychiatric disorders in patients with T2D.

## Materials and methods

We conducted a comparative cross-sectional study of individuals with T2D who attended a multidisciplinary comprehensive care model at a tertiary care center (the Center of Comprehensive Care for the Patient with Diabetes) [21].

### Study population

We included patients over 18 years old, with less than ten years since T2D diagnosis, body mass index (BMI) ≤ 45 kg/m2, without disabling complications, non-smokers or patients with substance use disorder, and without a psychotic psychiatric disorder.

### Procedures

Diagnosis of T2D was established according to the American Diabetes Association (ADA) criteria (HbA1c ≥ 6.5% or fasting plasma glucose ≥ 126 mg/dL or random plasma glucose ≥ 200 mg/dL with symptoms) [22]. Clinical characteristics (sex, age, time since T2D diagnosis, psychiatric comorbidity, and psychiatric treatment) and metabolic and anthropometric parameters (HbA1c, lipid profile, BMI, waist circumference, and body-fat percentage) were registered. Insulin resistance was calculated using the METS-IR score. This indirect method combines non-fasting laboratory values and anthropometric measurements to assess insulin sensitivity and detect insulin resistance in the Mexican population. The accuracy and diagnostic usefulness of the METS-IR set a cut-off point for insulin resistance at > 50.39 [23], which has been shown to identify insulin resistance in the Mexican population. This threshold was selected because it provides a clinically meaningful classification of individuals with and without insulin resistance, facilitating the interpretation of descriptive and stratified analyses. Psychiatric evaluation included a clinical interview by liaison psychiatrists and the application of the Mini International Neuropsychiatric Interview (M.I.N.I.) 5.0.0 Spanish version [24, 25], which is a short diagnostic structured interview to identify 16 psychiatric disorders according to the Diagnostic and Statistical Manual IV-TR edition (DSM-IV-TR) classification [26]. The Hospital Anxiety and Depression Scale (HADS) [27]: the accuracy and diagnostic usefulness of the Mexican version set a cut-off point for anxiety ≥ 8 (α-Cronbach 0.89, Kappa 0.68) and for depression ≥ 7 (α-Cronbach 0.72, Kappa 0.73) [28]. Psychiatric treatment included selective serotonin reuptake inhibitors (SSRIs), serotonin and norepinephrine reuptake inhibitors (SNRIs), benzodiazepines, hypnotics, and adjunctive treatment with atypical antipsychotics.

Blood samples were taken for measurement of lipid profile (using colorimetric methods, SYNCHRON CX System), HbA1c (using HPLC method, Bio-Rad Variant II Turbo HbA1c Kit 2), and body composition, and the percentage of fat mass was assessed by bioimpedance (body composition analyzer JAWON medical ioi353).

### Statistical analysis

Normality was assessed using the Kolmogorov-Smirnov test. We present the data with the median and the interquartile range. Categorical variables are reported in frequencies and percentages. The comparison between categorical variables was assessed using the χ2 test. Continuous variables were compared with the Mann-Whitney *U* test. We used STATA 14.0 (StataCorp. 2014. *Stata Statistical Software: Release 14*. College Station, TX: StataCorp LP). To determine whether the observed associations were independent of potential confounders, multivariable logistic regression models were constructed for affective, anxiety, and eating disorders. The models adjusted for age, glycemic control, adiposity, and other relevant metabolic variables. In addition, an interaction term between sex and METS-IR was included to formally assess effect modification.

### Power estimation

A post-hoc power analysis was performed using G*Power software (v. 3.1.9.7) to evaluate the sample adequacy for the multivariable logistic regression model. Considering the total sample size (*n* = 4699), a two-tailed significance level of alpha = 0.05, a baseline outcome prevalence of 45%, and adjusting for the remaining covariates in the model R^2^= 0.04, the sample of 4,699 patients yields a statistical power (1 - \beta) = 1.00 (100%) to detect an Odds Ratio 1.36 (OR females/OR males) per one standard deviation increase in the Mets-IR index.

## Results

Our sample included 4699 subjects with T2D; 57.2% were females, with a median age of 57 years (49–64), a median of 1 year (0–3) since T2D diagnosis, and a median HbA1c of 7.3% (6.1–9.8). The median METS-IR was 48.2 (42.1–55.2), and 42.4% of the total sample had a score compatible with insulin resistance. Affective disorders were identified in 21.7%, anxiety disorders in 22.3%, and eating disorders in 25.2%. In comparative analysis, patients with METS-IR ≥ 50 were associated with lower age (54 years (47–62) vs. 58 years (51–65); *p* < 0.001), worse metabolic parameters, and higher psychopathology, highlighting eating disorders (33.1% vs. 19.4%, *p* < 0.001), and psychiatric treatment (30% vs. 27.1%, *p* = 0.030) (Table 1). According to sex-based analysis, males were reported younger than females (53 years (45–61) vs. 56 years (48–61); *p* < 0.001), insulin resistance was higher in males than in females (48.9 [42.9–55.9] vs. 47.6 [41.4–54.5], *p* < 0.001), and metabolic parameters showed mixed results; HbA1c and fasting glucose were higher in males than in females (7.8 [6.2–10.2] vs. 7 [6-9.3], *p* < 0.001), and lipid profile was worse in females. Our most interesting results were in body composition, including body-fat percentage; females showed higher parameters than males (38.5 [35.2–41.9] vs. 31.1 [27.5–34.5]; *p* < 0.001), even though waist circumference and METS-IR were higher in males than females. All psychiatric disorders and psychiatric prescriptions were higher in females than in males (Table 2).

**Table 1:** Sociodemographic, metabolic, and psychiatric characteristics. Comparative analysis of patients with and without insulin resistance

Variable	Total *n* = 4699	Mets-IR < 50 *n* = 2708	Mets-IR ≥ 50 *n* = 1991	*p* value
Sex n (%)				
Females	2689 (57.2)	1616 (59.5)	1073 (53.9)	< 0.001
Males	2010 (42.8)	1092 (40.3)	918 (46.1)	< 0.001
Age (yr) Median (IQR)	57 (49–64)	58 (51–65)	54 (47–62)	< 0.001
Time since T2D diagnosis (yr)	1 (0–3)	1 (0–3)	1 (0–3)	0.133
SBP mmHg Median (IQR)	122 (114–130)	121 (112–130)	124 (117–132)	< 0.001
DBP mmHg Median (IQR)	76 (72–80)	75 (70–80)	78 (73–82)	< 0.001
Glucose mg/dL Median (IQR)	126 (101–177)	117 (97–156)	140 (109–202)	< 0.001
HbA1c (%) Median (IQR)	7.3 (6.1–9.8)	7 (6-9.3)	7.8 (6.3–10.2)	< 0.001
Total-cholesterol (mg/dL) Median (IQR)	183 (157–211)	184 (158–212)	182 (157–209)	0.120
LDL-cholesterol (mg/dL) Median (IQR)	112 (89–135)	114 (92–137)	109 (87–132)	< 0.001
HDL-cholesterol (mg/dL) Median (IQR)	42 (36–49)	45 (39–52)	38 (33–43)	< 0.001
Non-HDL-cholesterol (mg/dL) Median (IQR)	140 (114–166)	138 (112–163)	143 (119–169)	< 0.001
Triglycerides (mg/dL) Median (IQR)	167 (120–236)	146 (108–198)	200 (148–287)	< 0.001
Creatinine mg/dL Median (IQR)	0.7 (0.6–0.9)	0.7 (0.6–0.8)	0.7 (0.6–0.8)	0.062
Uric acid mg/dL Median (IQR)	5.2 (4.3–6.2)	5.1 (4.2-6)	5.5 (4.6–6.4)	< 0.001
ALT Median (IQR)	25 (18–38)	22 (16–32)	31 (21–48)	< 0.001
AST Median (IQR)	21 (16–28)	20 (16–25)	23 (17–32)	< 0.001
GGT Median (IQR)	30 (21–47)	26 (19–41)	35 (24–54)	< 0.001
Body mass index (kg/m^2^) Median (IQR)	29.1 (26-32.5)	26.6 (24.5–28.8)	33 (30.6–36.2)	< 0.001
Waist circumference (cm) Median (IQR)				
Females	97 (89–105)	92 (85.5–98)	106 (99–114)	< 0.001
Males	100 (93–108)	95 (89–100)	108 (101–116)	< 0.001
Body-fat percentage Median (IQR)				
Females	38.5 (35.2–41.9)	36.4 (34-38.9)	42.2 (39.7–44.5)	< 0.001
Males	31.1 (27.5–34.5)	28.5 (25.1–31.3)	34.1 (31.5–36.6)	< 0.001
Any psychiatric disorder				
Affective Disorder	1018 (21.7)	578 (21.3)	440 (22.1)	0.544
Anxiety Disorder	1049 (22.3)	586 (21.6)	463 (23.3)	0.194
Eating Disorder	1183 (25.2)	524 (19.4)	659 (33.1)	< 0.001
HADSd	5 (3–8)	5 (3–8)	6 (3–8)	0.024
HADSa	7 (5–10)	7 (4–10)	7 (5–11)	0.002
Psychiatric treatment	1333 (28.4)	735 (27.1)	598 (30)	0.030

Abbreviations: IQR, interquartile range; T2D, type 2 diabetes; SBP, Systolic Blood Pressure; DBP, Diastolic Blood Pressure; HbA1c, glycated hemoglobin; LDL, Low Density Lipoprotein; HDL, High-Density Lipoprotein; Non-HDL, Non-High Density Lipoprotein; HADS, Hospital Anxiety and Depression Scale

**Table 2:** Sociodemographic, metabolic, and psychiatric characteristics. Comparative analysis by sex

Variable	Total *n* = 4699	Females *n* = 2689	Males *n* = 2010	*p* value
Age (yr) Median (IQR)	57 (49–64)	57 (50–64)	55 (48–63)	< 0.001
Time since T2D diagnosis (yr)	1 (0–3)	1 (0–3)	1 (0–3)	0.250
SBP mmHg Median (IQR)	122 (114–130)	121 (112–130)	124 (116–132)	< 0.001
DBP mmHg Median (IQR)	76 (72–80)	76 (71–80)	77 (72–82)	< 0.001
Glucose mg/dL Median (IQR)	126 (101–177)	122 (98–168)	133 (106–190)	< 0.001
HbA1c (%) Median (IQR)	7.3 (6.1–9.8)	7 (6-9.3)	7.8 (6.2–10.2)	< 0.001
Total-cholesterol (mg/dL) Median (IQR)	183 (157–211)	187 (161–214)	178 (153–205)	< 0.001
LDL-cholesterol (mg/dL) Median (IQR)	112 (89–135)	114 (93–138)	108 (85–130)	< 0.001
HDL-cholesterol (mg/dL) Median (IQR)	42 (36–49)	44 (38–51)	38 (33–45)	< 0.001
Non-HDL-cholesterol (mg/dL) Median (IQR)	140 (114–166)	141 (116–167)	139 (113–164)	0.110
Triglycerides (mg/dL) Median (IQR)	167 (120–236)	161 (119–225)	174 (122–253)	< 0.001
Creatinine mg/dL Median (IQR)	0.7 (0.6–0.9)	0.6 (0.5–0.7)	0.8 (0.7–0.9)	< 0.001
Uric acid mg/dL Median (IQR)	5.2 (4.3–6.2)	4.9 (4.1–5.8)	5.8 (4.8–6.7)	< 0.001
ALT Median (IQR)	25 (18–38)	23 (16–36)	28 (20–42)	< 0.001
AST Median (IQR)	21 (16–28)	20 (16–28)	21 (17–28)	0.001
GGT Median (IQR)	30 (21–47)	27 (19–47)	34 (24–51)	< 0.001
Body mass index (kg/m2) Median (IQR)	29.1 (26-32.5)	29.1 (26.1–32.9)	28.9 (25.9–31.9)	0.028
Waist circumference (cm) Median (IQR)	98.7 (91–107)	97 (89–105)	100 (93–108)	< 0.001
Body-fat percentage Median (IQR)	35.3 (31-39.7)	38.5 (35.2–41.9)	31.1 (27.5–34.5)	< 0.001
Mets-IR	48.2 (42.1–55.2)	47.6 (41.4–54.5)	48.9 (42.9–55.9)	< 0.001
Affective Disorder	1018 (21.7)	747 (27.8)	271 (13.5)	< 0.001
Anxiety Disorder	1049 (22.3)	697 (25.9)	352 (17.5)	< 0.001
Eating Disorder	1183 (25.2)	778 (28.9)	405 (20.1)	< 0.001
HADSd	5 (3–8)	6 (3–8)	5 (3–8)	< 0.001
HADSa	7 (5–10)	8 (5–11)	7 (4–9)	< 0.001
Psychiatric treatment	1333 (28.4)	937 (34.9)	396 (19.7)	< 0.001

Abbreviations: IQR, interquartile range; T2D, type 2 diabetes; SBP, Systolic Blood Pressure; DBP, Diastolic Blood Pressure; HbA1c, glycated hemoglobin; LDL, Low Density Lipoprotein; HDL, High-Density Lipoprotein; Non-HDL, Non-High Density Lipoprotein; HADS, Hospital Anxiety and Depression Scale

For our final analysis, we grouped by METS-IR ≥ 50 and sex. In the females subgroup with METS-IR ≥ 50, a higher proportion of affective (30.5% vs. 26%, *p* = 0.011), anxiety (28% vs. 24.5%, *p* = 0.040), and eating disorders (38.3% vs. 22.7%, *p* < 0.001) were identified compared to the METS-IR < 50 subgroup; in the males group, only eating disorders showed a statistical difference (14.4% vs. 27%, *p* < 0.001). Nonetheless, for both females and males, HADS scores in the METS-IR ≥ 50 subgroups were higher than the METS-IR < 50 subgroups (Table 3). To determine whether the observed associations were independent of potential confounders, four multiple logistic regression models were constructed to evaluate the association between the sex-insulin resistance interaction and four distinct psychopathological outcomes (any psychiatric disorder, anxiety, affective and eating disorders), adjusting for age, body fat percentage, and glycated hemoglobin levels (Table 4). All four models demonstrated robust overall statistical significance (Likelihood Ratio χ^2^ omnibus tests, *p* < 0.001). For eating disorders (Model 1), a significant interaction effect emerged. Compared to the baseline reference group of males without insulin resistance, males with insulin resistance (males/Mets-IR ≥ 50) exhibited a 50% increase in the odds of eating disorders (OR = 1.50, 95%CI [1.17–1.93]; *p* = 0.001). The peak risk profile was observed among females with insulin resistance (OR = 1.85, 95%CI [1.36–2.52]; *p* < 0.001), whereas females without insulin resistance did not significantly differ from the reference group (*p* = 0.102). For affective (Model 2) and anxiety disorders (Model 3), the odds architecture was predominantly driven by male sex. In the affective disorders model, females without insulin resistance experienced over a twofold increase in odds (OR = 2.27, 95%CI [1.78–2.89]; *p* < 0.001), which was further intensified by comorbid insulin resistance (OR = 2.66, 95%CI [1.95–3.63]; *p* < 0.001). A similar pattern was observed for anxiety disorders (Model 3), where the subgroup of females with insulin resistance demonstrated the greatest relative risk (OR = 1.77, 95%CI [1.31–2.39]; *p* < 0.001). Regarding the adjusted covariates, older age acted as a consistent, marginal, yet highly significant protective factor across all four models (OR range= [0.97–0.98], *p* < 0.001). Finally, elevated glycated hemoglobin levels selectively increased the odds of experiencing affective disorders (OR = 1.07, 95%CI [1.04–1.10]; *p* < 0.001) and any psychiatric disorder (OR = 1.04, 95%CI [1.01–1.06]; *p* = 0.004).

**Table 3:** Sociodemographic, metabolic, and psychiatric characteristics. Comparative analysis by sex and insulin resistance

Variable	Females *n* = 2689			Males *n* = 2010		
	Mets-IR < 50 *n* = 1616	Mets-IR ≥ 50 *n* = 1073	*p* value	Mets-IR < 50 *n* = 1092	Mets-IR ≥ 50 *n* = 918	*p* value
Age (yr) Median (IQR)	59 (51–65)	56 (48–61)	< 0.001	57 (49–65)	53 (45–61)	< 0.001
Time since T2D diagnosis (yr)	1 (0–3)	1 (0–3)	0.299	1 (0–3)	1 (0–3)	0.113
SBP mmHg Median (IQR)	120 (111–130)	122 (115–130)	< 0.001	122 (114–130)	125 (119–134)	< 0.001
DBP mmHg Median (IQR)	74 (70–79)	77 (73–81)	< 0.001	76 (71–80)	79 (75–83)	< 0.001
Glucose mg/dL Median (IQR)	113 (95–149)	136 (107–196)	< 0.001	123 (101–170)	145 (113–209)	< 0.001
HbA1c (%) Median (IQR)	6.8 (5.9–8.7)	7.5 (6.3–10)	< 0.001	7.4 (6.1–10)	8.2 (6.5–10.5)	< 0.001
Total-cholesterol (mg/dL) Median (IQR)	189 (163–216)	184 (159–212)	0.019	176 (152–204)	178 (156–206)	0.242
LDL-cholesterol (mg/dL) Median (IQR)	117 (94–139)	112 (91-134.5)	< 0.001	111 (88–132)	104.5 (84–128)	< 0.001
HDL-cholesterol (mg/dL) Median (IQR)	48 (42–54)	40 (35–46)	< 0.001	42 (37–48)	35 (31–40)	< 0.001
Non-HDL-cholesterol (mg/dL) Median (IQR)	140 (114–166)	143 (118–171)	0.010	134 (109–161)	144 (120–168)	< 0.001
Triglycerides (mg/dL) Median (IQR)	145 (109–193)	193 (142–264)	< 0.001	147 (108–205)	216 (154–325)	< 0.001
Creatinine mg/dL Median (IQR)	0.7 (0.6–0.8)	0.6 (0.5–0.7)	0.469	0.8 (0.7–0.9)	0.8 (0.7–0.9)	0.005
Uric acid mg/dL Median (IQR)	4.8 (4-5.7)	5.1 (4.3–5.9)	< 0.001	5.5 (4.6–6.5)	5.9 (5.0–7)	< 0.001
ALT Median (IQR)	21 (15–31)	28 (18–45)	< 0.001	24 (17–33)	33 (24–51)	< 0.001
AST Median (IQR)	20 (16–26)	22 (17–32)	< 0.001	20 (16–25)	23 (18–32)	< 0.001
GGT Median (IQR)	24 (17–38)	31 (22–49)	< 0.001	29 (21–44)	39 (28-58.5)	< 0.001
Body-fat percentage Median (IQR)	36.4 (34-38.9)	42.2 (39.7–44.5)	< 0.001	28.5 (25.1–31.3)	34.1 (31.5–36.6)	< 0.001
Affective Disorder	420 (26)	327 (30.5)	0.011	158 (14.5)	113 (12.3)	0.154
Anxiety Disorder	396 (24.5)	301 (28)	0.040	190 (17.4)	162 (17.6)	0.896
Eating Disorder	367 (22.7)	411 (38.3)	< 0.001	157 (14.4)	248 (27)	< 0.001
HADSd	5 (3–8)	6 (3–9)	0.001	5 (3–7)	5 (3–8)	0.018
HADSa	7 (5–11)	8 (5–11)	0.006	6 (4–9)	7(4–10)	< 0.001
Psychiatric treatment	525 (32.5)	412 (38.4)	0.002	210 (19.2)	186 (20.3)	0.563

Abbreviations: IQR, interquartile range; T2D, type 2 diabetes; SBP, Systolic Blood Pressure; DBP, Diastolic Blood Pressure; HbA1c, glycated hemoglobin; LDL, Low Density Lipoprotein; HDL, High-Density Lipoprotein; Non-HDL, Non-High Density Lipoprotein; HADS, Hospital Anxiety and Depression Scale

**Table 4:** Multiple logistic regression models predicting psychiatric disorders by sex and insulin resistance status (*N* = 4699)

Predictor Variables	Model 1: Eating disorders		Model 2: Affective disorders		Model 3: Anxiety disorders		Model 4: Any psychiatric disorder		
	OR	95% CI	OR	95% CI	OR	95% CI	OR	95% CI	
**Sex × Insulin Resistance Interaction**									
Males / Mets-IR ≥ 50	1.50**	[1.17, 1.93]	0.78	[0.59, 1.03]	0.95	[0.73, 1.22]	1.11	[0.90, 1.37]	
Females / Mets-IR < 50	1.22	[0.96, 1.56]	2.27***	[1.78, 2.89]	1.55***	[1.23, 1.95]	1.76***	[1.45, 2.14]	
Females / Mets-IR ≥ 50	1.85***	[1.36, 2.52]	2.66***	[1.95, 3.63]	1.77***	[1.31, 2.39]	2.40***	[1.85, 3.11]	
**Adjusted Covariates**									
Age (years)	0.97***	[0.97, 0.98]	0.98***	[0.98, 0.99]	0.98***	[0.97, 0.99]	0.97***	[0.96, 0.98]	
Body-Fat Percentage	1.05***	[1.03, 1.07]	1.00	[0.98, 1.01]	1.00	[0.99, 1.02]	1.02**	[1.00, 1.03]	
HbA1c (%)	1.01	[0.98, 1.04]	1.07***	[1.04, 1.10]	1.00	[0.97, 1.02]	1.04**	[1.01, 1.06]	
**Model Fit Indices**									
Model Wald χ² (df = 6)	275.42		208.12		95.14		311.33		
McFadden’s Pseudo R²	0.0519		0.0424		0.0191		0.0482		

* *p* < 0.05, ** *p* < 0.01, *** *p* < 0.001 (computed from the Wald z-test values)

## Discussion

The relationship between T2D and psychiatric disorders has been explored for many years, and the CNS, like other insulin-sensitive tissues, has emerged as a focus of investigation, given the high expression of insulin receptors in brain regions involved in cognition, mood, and feeding behavior [29–31]. This study examined the association between the most prevalent psychiatric disorders (anxiety, affective, and eating disorders) and central insulin resistance estimated using the METS-IR index in T2D subjects, with a focus on sex differences. Our findings reveal a pronounced sex-dependent dimorphism: while elevated METS-IR ≥ 50 was significantly associated with eating disorders in both sexes, its association with affective (OR = 2.66) and anxiety disorders (OR = 1.77) was uniquely pronounced in females, showing no significant association in males.

These clinical observations align with preclinical evidence demonstrating that central insulin resistance impairs CNS function through four distinct molecular pathways. First, alterations in insulin receptor signaling and its PI3K/Akt/mTOR pathway have been associated with changes in ATP synthesis and mitochondrial metabolism, thereby being linked to cellular functions such as synaptic plasticity, cholesterol synthesis, neuronal survival, and neurotransmitter trafficking. Also, alterations in the Shc/Ras/ERK/MAPK pathway have been linked to cell growth and proliferation. The nuclear factor kappa-light-chain-enhancer of activated B cell (NF-κB) pathway has been associated with increased expression of proinflammatory cytokines (IL-1, IL-6, TNF-α), and the activation of c-Jun N-terminal kinase has been reported to accompany disruption of brain insulin signaling, as a consequence of the accumulation of damaging free fatty acids and ceramides in the brain because of high-fat diet intake. Also, mitochondrial metabolism and its homeostasis have been implicated in hypothalamic functions through AgRP and POMC neuronal activity; finally, due to the high sensitivity of the brain to damage from oxidative stress, insulin resistance has been associated with altered antioxidant expression in the CNS [32]. These changes suggest biological pathways through which central insulin signaling may be related to feeding behavior, insulin sensitivity in peripheral tissues, glucose homeostasis, and fat storage throughout the body [4]. This neurobiological framework provides a compelling rationale for the marked sex dimorphism observed in our results. Importantly, the interplay among these pathways provides a plausible neurobiological basis for the female vulnerability observed in our cohort. Estrogen signaling is known to interact synergistically with central insulin receptors, particularly within the hippocampus, amygdala, and hypothalamus—regions critical for emotional regulation and affective control. In females, central insulin resistance and impaired PI3K/Akt signaling disrupt this protective crosstalk, exacerbating neuroinflammation via the NF-kB pathway and impairing synaptic plasticity to a greater degree than in males. Furthermore, sex-specific differences in hypothalamic AgRP/POMC neuronal sensitivity and central ceramide accumulation may heighten female vulnerability to metabolic stress, ultimately translating into the significantly higher odds for affective (OR = 2.66) and anxiety disorders (OR = 1.77) observed in women with METS-IR ≥ 50, a pattern notably absent in their male counterparts [33]. However, the exact molecular mechanism underlying the relationship between insulin resistance and neuropsychiatric disorders is not currently well elucidated. Genetic studies identified that phenotypes associated with insulin resistance have a genetic correlation with Alzheimer disease, autism spectrum disorder, obsessive-compulsive disorder, anorexia nervosa, attention-deficit/hyperactivity disorder, bipolar disorder, major depressive disorder, and schizophrenia; however, results suggest different clusters of neuropsychiatric disorders, in which the genetics of insulin resistance may have divergent pleiotropic effects [34, 35].

Epidemiologic studies of recently diagnosed T2D patients found that 40.1% have non-psychotic psychiatric comorbidity [36]. An umbrella review and meta-analysis of 32 systematic reviews that included subjects with T2D reported prevalence estimates ranging from 5% to 22%, depending on the specific psychiatric disorder [37]. In a population-based cohort study of 1,883,198 Danish citizens, T2DM was associated with eating disorders, schizophrenia spectrum, obsessive-compulsive disorder, autism spectrum disorder, attention-deficit/hyperactivity disorder, substance use disorder, major depressive disorder, bipolar disorder, anxiety disorder, and personality disorder. For most psychiatric disorders, associations were strongest in females [38]. These results align with our findings, in which eating disorders are the principal psychiatric disorder associated with T2D, and all non-psychotic psychiatric disorders are more prevalent in females than in males. Some studies in subjects without T2D have associated insulin resistance with psychiatric disorders, and the principal and strongest association is for neurodegenerative disorders [3, 39], but there is a lack of information about other disorders. A meta-analysis about insulin resistance in patients with and without depression included 70 studies and identified an increase in the HOMA-IR index only in patients with atypical depression; they consider it a metabolic phenotype for depression, and they propose insulin resistance as a state biomarker in acute depression [40]. We identified that not all patients with psychiatric disorders have an insulin resistance index affected (METS-IR ≥ 50); however, subgroups of patients with higher insulin resistance exhibited greater psychiatric symptom severity, higher frequency of any psychiatric disorder, and higher risk for any psychiatric disorder, except affective and anxiety disorders in the male subgroup. Our results are consistent with a recent Egyptian case–control study that included 60 participants and compared 3 groups: insulin-resistant T2D subjects, insulin-resistant non-T2D subjects, and non-insulin-resistant controls; they reported metabolic parameters similar to ours (45% of the participants are found to have insulin resistance (HOMA IR > 2.9), while HbA1c range is beow 7%); however, they found higher rates of depression and anxiety symptoms in insulin-resistant groups, with 75% of insulin-resistant T2D and 60% of insulin-resistant non-T2D depressed, vs. 35% in controls (*p* = 0.036). Anxiety showed a similar gradient, and anxiety correlated with higher HOMA-IR values [41]. We reported a minor prevalence of depression and anxiety disorders, and this difference may be explained by the fact that our results report formal diagnosis with the gold standard (psychiatrist) and not only scale measurements. From a treatment perspective, we identified a higher prescription rate of psychopharmacologic treatment in insulin resistance subgroups, and there is evidence of differences in therapeutic response [42]. UK Biobank primary-care data (30919 MDD cases) showed that > 50% had insulin-resistance–related conditions; these patients required more antidepressant classes, had longer treatment duration, and were more likely to be non-responders or treatment-resistant [31].

Insulin resistance and eating disorders (bulimia nervosa and binge eating disorder as principal) have consistently been associated [43]. Insulin regulates both metabolism and brain reward systems, so changes in insulin sensitivity may influence appetite, mood, and eating behavior [44]. At the same time, disordered eating can in turn worsen insulin resistance or glycemic control. Our results showed that eating disorders are associated with insulin resistance in all groups, with higher odds that were significantly elevated in males with insulin resistance and reached the highest magnitude in females. A meta-analysis that examines the association between eating disorders and insulin sensitivity included 12 studies (3264 subjects for bulimia nervosa and binge eating disorder). The main finding was that a statistically significant association exists between bulimia nervosa/binge eating disorder and insulin sensitivity (moderate pooled estimate of the mean standardized effect sizes (g = − 0.57; 95% CI: −0.80, − 0.34) [45]. We highlight the association between the highest body-fat percentage and psychiatric disorders in the female group, despite the other variables of adiposity being higher in the male group. A recent Italian systematic review examined the interplay among insulin resistance, affective dysregulation, and disordered eating behaviors in the context of obesity [46].

The higher prevalence of psychiatric disorders among females with insulin resistance is likely to reflect an interplay of shared biological pathways, condition-specific clustering, and unique psychosocial burdens. Biologically, insulin resistance has been linked to brain circuits related to mood and reward, stress hormones, and inflammation—factors already deeply implicated in female-predominant disorders like depression and anxiety [47, 48]. This pattern may also be influenced by the high prevalence of conditions such as polycystic ovary syndrome (PCOS), gestational diabetes, and obesity in females, all of which carry a significant insulin resistance burden and are linked to increased psychiatric morbidity [49, 50]. Beyond biology, gendered psychosocial stressors—including body image concerns, fertility issues, weight stigma, and the psychological demands of insulin therapy—may further contribute to these observed associations in females [48, 51]. Beyond the interaction between sex and METS-IR, our multivariable models revealed distinct independent contributions of metabolic covariates depending on the psychiatric outcome. Notably, body fat percentage was independently associated with increased odds of eating disorders (OR = 1.05; 95%CI [1.03–1.07]), whereas HbA1c showed no significant association with this diagnostic category. This suggests that the psychological and somatic burden of dysfunctional eating behaviors in T2D is more tightly coupled with adiposity-driven signaling and peripheral body composition than with chronic glycemic control per se. Conversely, elevated HbA1c levels independently increased the odds of affective disorders (OR = 1.07; 95%CI: 1.04–1.10), while body fat percentage remained non-significant. Sustained hyperglycemia and glycemic variability are known to induce systemic glucotoxicity, microvascular damage, and advanced glycation end-product (AGE) accumulation within brain microvasculature, leading to neuroinflammation and compromised monoaminergic transmission in limbic circuits. Thus, chronic systemic metabolic dysregulation (reflected by HbA1c) appears to exert a direct, cumulative neurotoxic effect on mood regulation pathways, independent of central insulin resistance and overall adiposity [51].

However, because of the cross-sectional design and the sample characteristics, our findings do not allow conclusions regarding temporality or causality, and longitudinal studies are needed to clarify the directionality and underlying mechanisms of these associations. We acknowledge that METS-IR measures peripheral rather than central insulin resistance, which could be considered a limitation. However, an increasing body of evidence indicates that peripheral metabolic dysfunction is tightly coupled with changes in brain insulin signaling.

## Conclusion

This study highlights important sex-specific differences in the association between insulin resistance and psychiatric comorbidities in patients with type 2 diabetes. Higher insulin resistance was associated with affective and anxiety disorders only among females, suggesting that sex modifies the relationship between insulin resistance and psychiatric outcomes. This study demonstrates a sex-specific association between insulin resistance, estimated by the METS-IR index, and psychiatric comorbidities in individuals with type 2 diabetes. Higher METS-IR was associated with affective and anxiety disorders among females, whereas in males, the association was primarily observed with eating disorders. Although the cross-sectional design precludes causal inference, these findings support the value of considering both metabolic status and sex when screening for psychiatric comorbidities in people with type 2 diabetes. Prospective studies are warranted to clarify the temporal and mechanistic relationships underlying these associations. These results emphasize the necessity of sex-stratified clinical approaches, suggesting that monitoring metabolic markers of insulin resistance could serve as valuable information to consider that females are at higher risk for some neuropsychiatric complications in T2D from recent diagnosis. Implementing integrated care models that address both metabolic and mental health is essential for improving clinical outcomes and the quality of life in patients with type 2 diabetes.

## Data Availability

The dataset supporting the conclusions of this article is available in the Figshare repository at https://doi.org/10.6084/m9.figshare.32809154(52) [52].
